# Lipid metabolism-related gene signature predicts prognosis and depicts tumor microenvironment immune landscape in gliomas

**DOI:** 10.3389/fimmu.2023.1021678

**Published:** 2023-02-13

**Authors:** Junhong Li, Shuxin Zhang, Siliang Chen, Yunbo Yuan, Mingrong Zuo, Tengfei Li, Zhihao Wang, Yanhui Liu

**Affiliations:** ^1^ Department of Neurosurgery, Chengdu Second People’s Hospital, Chengdu, Sichuan, China; ^2^ Department of Neurosurgery, West China Hospital of Sichuan University, Chengdu, Sichuan, China

**Keywords:** lipid metabolism, glioma, tumor microenvironment, immune, prognosis

## Abstract

**Background:**

Glioma is the most common primary brain tumor in adults and accounts for more than 70% of brain malignancies. Lipids are crucial components of biological membranes and other structures in cells. Accumulating evidence has supported the role of lipid metabolism in reshaping the tumor immune microenvironment (TME). However, the relationship between the immune TME of glioma and lipid metabolism remain poorly described.

**Materials and methods:**

The RNA-seq data and clinicopathological information of primary glioma patients were downloaded from The Cancer Genome Atlas (TCGA) and Chinese Glioma Genome Atlas (CGGA). An independent RNA-seq dataset from the West China Hospital (WCH) also included in the study. Univariate Cox regression and LASSO Cox regression model was first to determine the prognostic gene signature from lipid metabolism-related genes (LMRGs). Then a risk score named LMRGs-related risk score (LRS) was established and patients were stratified into high and low risk groups according to LRS. The prognostic value of the LRS was further demonstrated by construction of a glioma risk nomogram. ESTIMATE and CIBERSORTx were used to depicted the TME immune landscape. Tumor Immune Dysfunction and Exclusion (TIDE) was utilized to predict the therapeutic response of immune checkpoint blockades (ICB) among glioma patients.

**Results:**

A total of 144 LMRGs were differentially expressed between gliomas and brain tissue. Finally, 11 prognostic LMRGs were included in the construction of LRS. The LRS was demonstrated to be an independent prognostic predictor for glioma patients, and a nomogram consisting of the LRS, IDH mutational status, WHO grade, and radiotherapy showed a C-index of 0.852. LRS values were significantly associated with stromal score, immune score, and ESTIMATE score. CIBERSORTx indicated remarkable differences in the abundance of TME immune cells between patients with high and low LRS risk levels. Based on the results of TIDE algorithm, we speculated that the high-risk group had a greater chance of benefiting from immunotherapy.

**Conclusion:**

The risk model based upon LMRGs could effectively predict prognosis in patients with glioma. Risk score also divided glioma patients into different groups with distinct TME immune characteristics. Immunotherapy is potentially beneficial to glioma patients with certain lipid metabolism profiles.

## Introduction

Glioma is the most common primary brain tumor in adults and accounts for more than 70% of brain malignancies (1). According to the latest 2021 World Health Organization (WHO) Classification of tumors of the central nervous system, gliomas are classified into 4 grades (1–4) and their diagnoses rely more on unique molecular biomarkers since which was first introduced in 2016 (2, 3). The grade 4 glioblastoma (GBM) represents the most lethal and malignant glioma with an incidence about 3.2 per 100000 population (4). GBMs are notorious for resistance to therapy. Despite surgical intervention and combined radio-chemotherapy, the median overall survival (OS) of patients with GBM is usually less than 2 years (5). Molecular therapies targeting oncogenic pathways or immune checkpoints in gliomas have been extensively studied, but very few concrete advances have been made to significantly improve patient outcome (6, 7).

Lipids, including phospholipids, fatty acids, triglycerides, sphingolipids, cholesterol, and cholesteryl esters, are crucial components of biological membranes and other structures in cells (8). Moreover, lipids are used in energy storge and metabolism and play important roles in cellular signaling pathway. Lipid metabolism dysregulation is among the most prominent metabolic alterations in cancer development (9). For example, tumor cells can increase lipogenesis, fatty acid (FA) uptake, and FA oxidation for energy production and lipid accumulation (10). Emerging evidence also indicates that cancer stem cells undergo lipid metabolism reprogramming, which helps maintain the properties of cancer stem cells (11, 12). Meanwhile, targeting the lipid metabolism regulating pathway has been regarded as a novel anti-cancer strategy (8).

Metabolism reprogramming occurs not only in cancer cell initiation and progression, but also in immune cells from tumor microenvironment (TME) (13, 14). Accumulating evidence has supported the role of lipid metabolism in shaping the TME. Recent studies revealed that lipid metabolism influences T cell differentiation, survival, and effector functions (15). Su et al. found accumulations of lipids in tumor-associated macrophages (TAMs) was crucial for their differentiation and function in tumor progression (16). Lipid metabolites also help modify the TME and affect the recruitment and of tumor-related immune cells (17). Kobayashi et al. reported that increasing lipid metabolism impaired the function of natural killer (NK) cells (18). Dysregulation of lipid metabolism in TME also influences the tumor-related immune response, which in most cases is presented as immunosuppressive effects (19). Nevertheless, the relationships between glioma and lipid metabolism remain largely unexplored.

With the rapid improvement of multiomic databases and tools that extract TME composition from bulk tumor transcriptomic data, we are now able to explore how dysregulation of lipid metabolism pathways associate with glioma progression and TME immune characteristics. In the current study, based on RNA-sequencing, we conducted a comprehensive and rigorous bioinformatic analysis to elucidate the prognostic ability of lipid metabolism-related genes (LMRGs) in patients with glioma, and disclosed its association with TME immune landscape.

## Materials and methods

### Data sources

The RNA-sequencing fragment per kilobase million (FPKM) transcriptome data and corresponding clinicopathological information of primary glioma patients were downloaded from The cancer Genome Atlas (TCGA, https://www.cancer.gov/) (20) and Chinese Glioma Genome Atlas (CGGA, http://www.cgga.org.cn/) (21), respectively. Recurrent gliomas and patients with incomplete survival data were excluding from the current research. A total of 666 patients from TCGA and 229 patients from CGGA were included in current study.

Another cohort from West China Hospital (WCH) which contains 78 patients with glioma were also included in the current research. ​After initial treatment of surgery, the patients were followed up every 3-6 months for evaluating prognosis. Overall survival (OS) was defined as the duration from the date of operation to death or the end of the observation period. The clinicopathological information of cohorts was list in **Table 1**.

**Table 1:** Clinicopathological characteristics of glioma patients in TCGA, CCGA, and WCH cohort.

Glioma Patients	TCGA cohort	CGGA cohort	WCH cohort
Sample size	666	229	78
Normal Brain	5	-	16
Age	46 (14-89)	43 (10-79)	46 (14-77)
Sex			
Female	282	87	31
Male	382	142	47
NA	2	0	0
Histology			
Astrocytoma	343	84	22
Oligodendroglioma	168	60	21
Glioblastoma	155	85	35
WHO Grade			
G2	216	94	29
G3	237	50	14
G4	155	85	35
NA	58	0	0
IDH mutation status			
Mutant	424	116	42
WT	237	112	36
NA	5	1	0
1p19q codeletion status			
Codel	168	54	19
Non-codeletion	491	172	44
NA	7	3	15
TERT promoter mutation status		-	
Mutant	341	-	30
WT	157	-	24
NA	168	-	24
MGMT promoter methylation status			
Methylated	473	99	36
Unmethylated	160	116	13
NA	33	14	29
ATRX mutation status		-	
Mutant	194	-	23
WT	461	-	53
NA	11	-	2

TCGA, The cancer Genome Atlas; CGGA, Chinese Glioma Genome Atlas; WCH, West China Hospital; WHO, World Health Organization; IDH, isocitrate dehydrogenase; TERT, telomerase reverse transcription; MGMT, O6-methylguanine-DNA methyltransferase; ATRX, alpha thalassemia/mental retardation syndrome X-Linked protein/gene; WT, wild; NA, not available.

### RNA extraction, sequencing, and differential analysis

Frozen glioma and adjacent brain tissue samples from West China Hospital were homogenized, and total RNA was isolated using Trizol reagent (Invitrogen, USA). After checking RNA quality, 2 μg RNA per sample was used as input material for the RNA sample preparations. mRNA was purified from total RNA by using poly-T oligo-attached magnetic beads. PCR products were purified (AMPure XP system). The mRNA was reverse-transcribed into a cDNA library and was sequenced on Illumina Novaseq S6000 platform to generate 150 bp paired-end reads. Clean reads were mapped to the hg19 genome and counted using STAR (2.6.0c). Differential expression analysis was conducted using R package limma. Genes with adjusted p value below 0.05 were defined as DEGs.

### Screening of lipid metabolism-related genes

The LMRGs were obtained from the Molecular Signature Database (MsigDB, v7.5.1, https://www.gsea-msigdb.org/) (22), including the following pathways: glycerophospholipid metabolism, adipocytokine signing pathway, PPAR signaling pathway, glycerolipid metabolism, regulation of lipolysis in adipocytes, fatty acid metabolism, arachidonic acid metabolism, sphingolipid metabolism, cholesterol metabolism, fatty acid degradation, ether lipid metabolism, steroid hormone biosynthesis, fatty acid elongation, fat digestion and absorption, biosynthesis of unsaturated fatty acids, steroid biosynthesis, linoleic acid metabolism, alpha-linolenic acid metabolism, primary bile acid biosynthesis (**Table S1**). The LMRGs were further filtered by intersecting with DEGs between glioma and brain tissue samples in the TCGA dataset.

### Prognostic lipid metabolism-related risk score construction and validation

An LMRGs-related risk score (LRS) was performed to establish a prognostic assessment method based on the expression of LMRGs. First, glioma patients from TCGA were randomly divided into a training group and a validation set with a ratio of 6:4. In the training group, the screened LMRGs were included in the univariate Cox regression to preliminarily filter for prognostic genes. Then least absolute shrinkage and selection operator (LASSO) Cox regression model was further used to select the strongest prognostic signature through minimizing the risk of over-fitting using “glmnet” R package. One hundred LASSO Cox-regression models were generated using different random number seeds. LMRGs with non-zero coefficients in over 50 models were selected to build a final Cox regression model. Finally, the LRS was calculated with the following algorithm:


$$LRS=\;\sum_{i=1}\beta_{i}*Exp_{i}$$

In the formula, *Exp* and *β* represent the expression level and coefficient of each prognostic LMRG in the final Cox regression model, respectively. The “survminer” R package was utilized to determine an optimal cutoff value to divide the specific cohort into two risk group. Based on the optimal cutoff value of LRS, glioma patients were divided into low-risk group (LRS value <optimal cutoff value) and high-risk group (LRS value ≥optimal cutoff value).

Next, the prognostic ability of LRS was verified in training group and validation group respectively, and patients from CGGA and WCH were used as external group for further validation. In each cohort, the patients were divided into two risk group using the surv_cutpoint function from the survminer package. The function set an optimal cutoff of LRS between its 10% and 90% percentile by maximizing the log-rank statistics. Patients with LRS below the cutoffs were stratified into LRS low risk groups, while those with LRS above the cutoffs were stratified into the LRS high risk groups. Time-dependent receiver operating characteristic (ROC) curves of the LRS were constructed at 1, 2, and 3 years.

### Construction and validation of nomogram for predicting prognosis

To understand the prognostic value of LRS in the context of other clinical and pathological factors. We conducted univariate and multivariate Cox regression analysis to screen for independent risk factors among a panel of variables including the LRS, age, sex, WHO grade, Karnofsky Performance Scale (KPS), IDH mutational status, 1p/19q codeletion status, chemotherapy, and radiotherapy. Variables with a p value <0.1 were included in multivariate Cox regression for further analysis, and variables with a p value <0.05 in multivariate Cox regression analysis were deemed as independent risk factors and were used to constitute the prognostic nomogram using the “rms” R package. Performance of the nomogram was assessed by calibration curve. Nomogram were built and evaluated in the TCGA, CGGA, and WCH cohort respectively.

### Functional analysis of DEGs between LRS high and low risk groups

Differentially expressed genes between LRS high- and low-risk groups with adjusted p value below 0.05, and log (Fold Change) greater than 0.5 or below -0.5 were defined as LRS-associated DEGs. Functional over-representation and Gene-Set Enrichment Analysis of DEGs between the LRS high- and low-risk groups were performed and visualized by using the “clusterProfiler” R package. Gene set variation analysis (GSVA) were conducted using the “GSVA” R package. Differentially expression of gene sets was conducted using R package limma. The KEGG, Gene ontology (GO), and HALLMARK gene sets curated in the msigdbr package were used as the gene set database for the functional analysis.

### Genetic alterations in LMRGs

Genetic alterations including deletion, amplification, and mutations of gliomas in the TCGA cohort were downloaded from cbioportal (https://www.cbioportal.org). We used the Genomic Identification of Significant Targets in Cancer (GISTIC) score data from the cbioportal to represent copy number variations. The mutational landscape of LRS low- and high-risk groups were presented using “maftools” R package.

### Immune-related analysis based on LRG signatures

Estimation of Stromal and Immune cells in Malignant Tumor tissues using Expression data (ESTIMATE, https://bioinformatics.mdanderson.org/) is a tool for predicting tumor purity and the presence of infiltrating stromal/immune cells based on gene expression data (23). Three scores included stromal score (presence of stroma in tumor tissue), immune score (infiltration of immune cells in tumor tissue), and ESTIMATE score (related to tumor purity) were generated through the algorithm. The tumor purity data were computed as described by D.Aran et al. with the formula (23): Tumour purity=cosine(0.6049872018 + 0.0001467884 × ESTIMATE score).

CIBERSORTx (https://cibersortx.stanford.edu/) provide an estimation of the abundances of member cell types in a mixed cell population by using gene expression data (24). Furthermore, correlations between risk groups and expression of immune check points (ICP) were analyzed.

### Therapeutic response prediction

Tumor Immune Dysfunction and Exclusion (TIDE, http://tide.dfci.harvard.edu/) was utilized to predict the immune checkpoint blockade (ICB) response in treating gliomas based on the functional status of cytotoxic T lymphocytes (25). In addition, TMZ sensitivity in treating glioma patients was assessed using “pRRophetic” R package (26).

### Statistical analysis

R software (version 3.6.1) and the above-mentioned package were used to handle the RNA-sequencing relevant data. K-M analysis was conducted to evaluate prognosis of specific glioma groups using log-rank test. A two-sided p<0.05 was regarded as statistically significant and * indicated p<0.05, whereas ** p<0.01, *** p<0.001, **** p<0.0001 in the current study.

### Ethics statement

This study was approved by the Ethical Committee of Sichuan University and conducted according to the principles expressed in the Declaration of Helsinki (Ethic number: 2018.569). All patients and their authorized trustees were informed before surgery and signed their informed consent to using their clinical data for research purposes.

## Results

### LMRGs determination in gliomas

**Figure 1:**
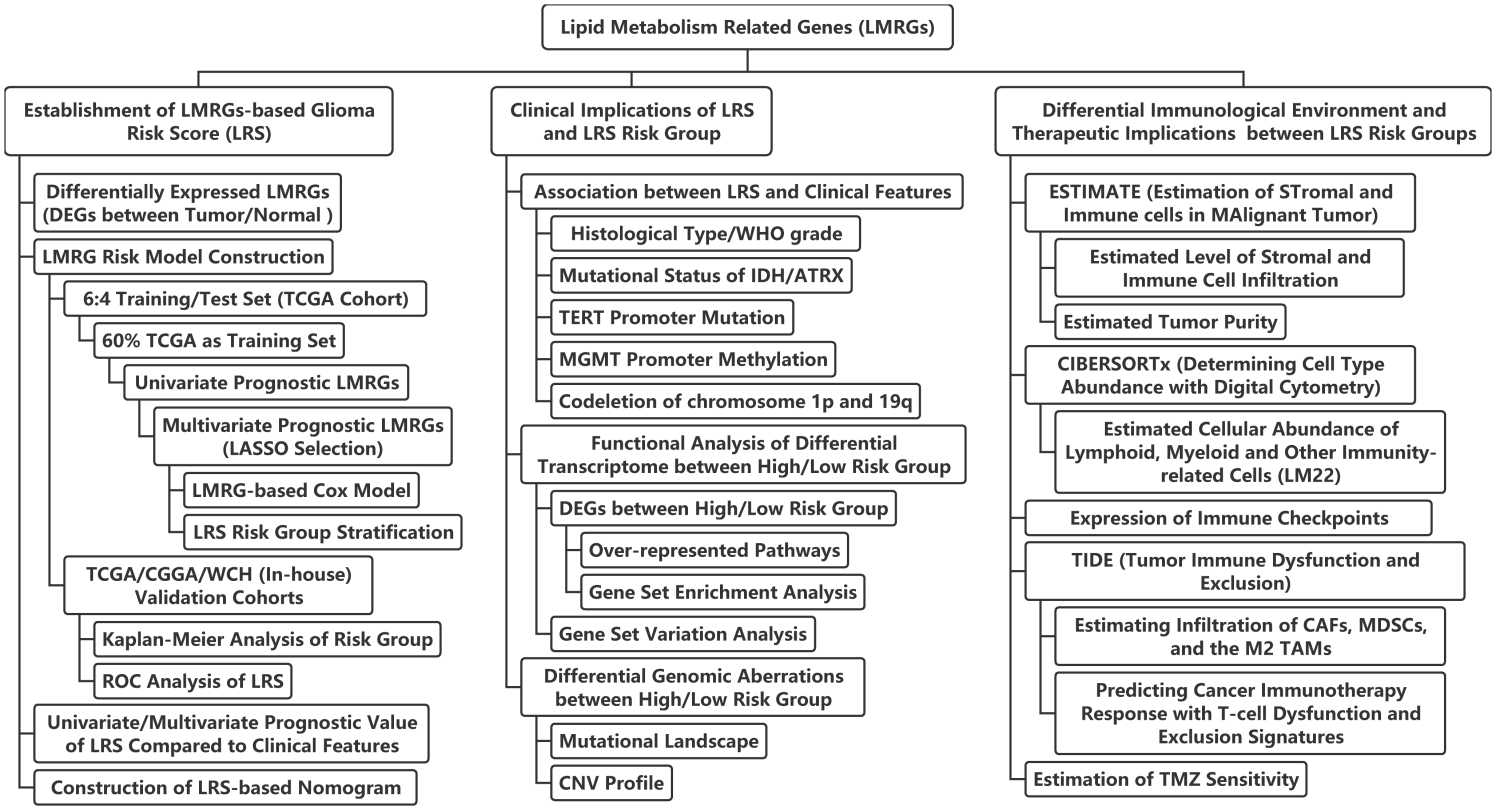
Flow chart of the current study. LASSO, least absolute shrinkage and selection operator; ROC, receiver-operator characteristics; IDH, isocitrate dehydrogenase; TERT, telomerase reverse transcription; MGMT, O6-methylguanine-DNA methyltransferase; ATRX, alpha thalassemia/mental retardation syndrome X-Linked protein/gene; CNV, copy-number variation; CAFs, cancer-associated fibroblasts; MDSCs, myeloid-derived suppressor cells; TAMs, tumor-associated macrophages; TMZ, temozolomide.

**Figure 2:**
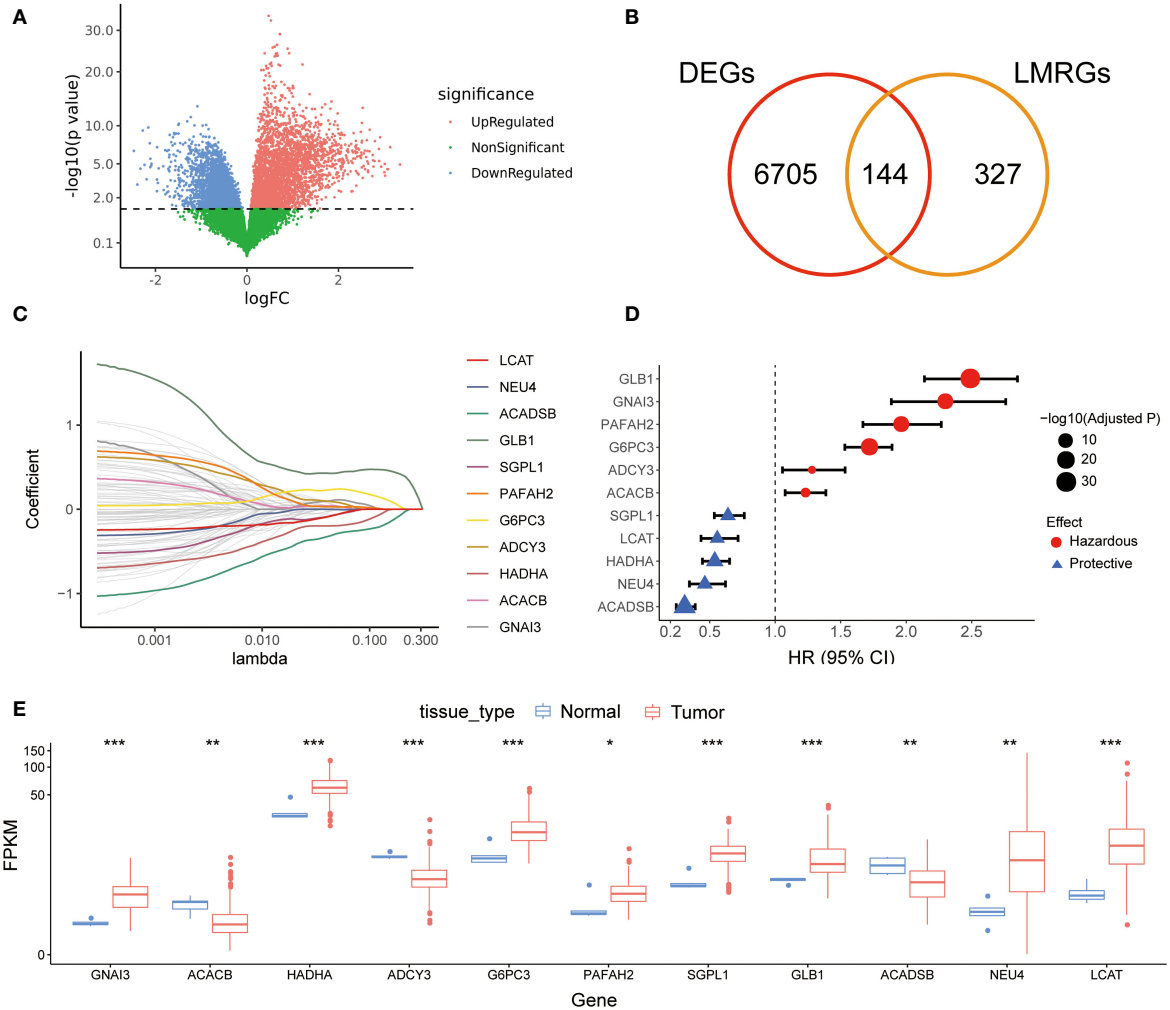
Screening of prognostic LMRGs. **(A)**, DEGs between gliomas and normal brains in TCGA cohort; **(B)** Venn diagram for screening common genes in DEGs and LMRGs; **(C)** LASSO Cox regression for screening reliable prognostic LMRGs; **(D)** Forest plot of 11 prognostic LMRGs; **(E)** Expression levels of 11 prognostic LMRGs in tumor tissues and normal brains. LMRG, lipid metabolism-related gene; DEG, different expression gene; LASSO, least absolute shrinkage and selection operator. * indicated p<0.05, whereas ** p<0.01, *** p<0.001 in the current study.

The workflow of the current study was illustrated in **Figure 1**. A total of 471 LMRGs were downloaded from the MSigDB (**Table S2**). We then identified 6849 DEGs between glioma and normal brain samples according to the cutoff of false discovery rate (FDR) <0.05 (**Figure 2A**; **Table S3**), including 3494 upregulated genes and 3355 downregulated genes. Among these DEGs, a total of 144 LMRGs were in overlap with genes curated in the lipid metabolism related gene sets in the MSigDB (**Figure 2B**).

### Development and validation of the prognostic lipid metabolism risk score

After randomly splitting the TCGA cohort into training and validation set at a ratio of 6:4, we conducted univariate Cox regression analysis using the 144 LMRGs. In the training set, we found 96 candidate LMRGs were significantly associated with patient survival. Next, these candidate genes were filtered using repeated LASSO regression. A total of 11 LMRGs (**Figure 2C**) were selected to build the lipid metabolism-related risk score (LRS). Among the 11 prognostic LMRGs, 5 of them predicted better prognosis while the other 6 predicted worse prognosis (**Figure 2D**). Expression levels of the 11 prognostic LMRGs in tumor tissues and normal brains were shown in **Figure 2E**.

**Figure 3:**
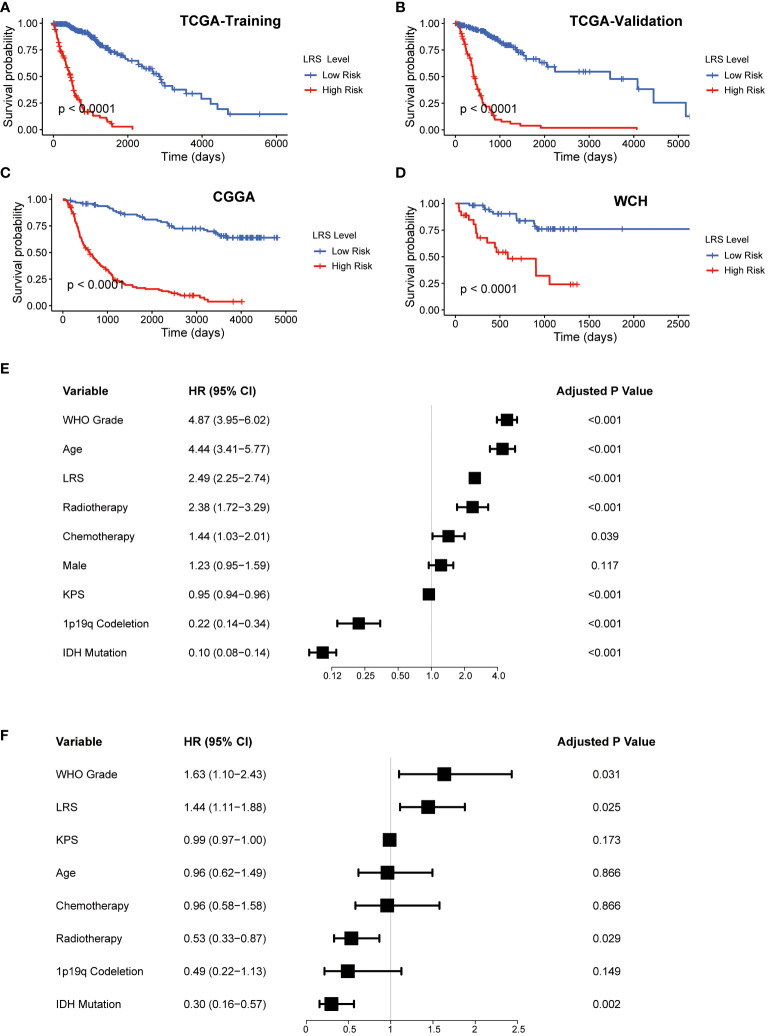
Prognostic value of LRS. **(A-D)**, K-M curves for assessing LRS in TCGA-training group, TCGA-validation group, CGGA, WCH cohort respectively; **(E)**, Univariate Cox regression of clinical variables in entire TCGA cohort; **(F)**, Multivariate Cox regression of clinical variables with a p value <0.1 in univariate Cox regression in entire TCGA. LRS, lipid metabolism risk score; K-M, Kaplan-Mier; TCGA, The cancer Genome Atlas; CGGA, Chinese Glioma Genome Atlas; WCH, West China Hospital.

**Figure 4:**
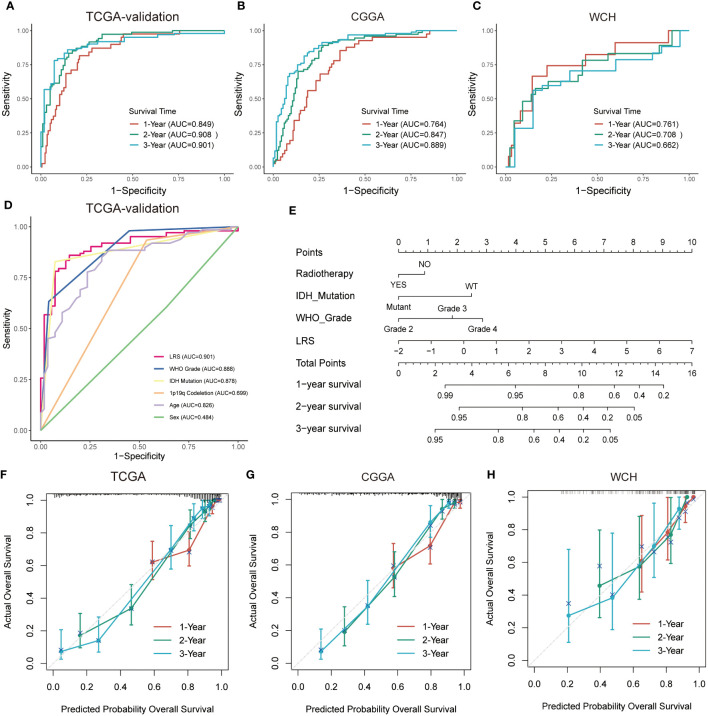
Prognostic predictive performance of LRS and nomogram construction. **(A–C)**, ROC curves for predicting 1-, 2-, 3-year overall survival in TCGA-validation group, CGGA, and WCH cohort; **(D)**, overall survival prediction based on clinicopathological variables in TCGA-validation cohort. **(E)**, Nomogram construction based on independent prognostic clinical variables; **(F–H)**, Calibration curve for assessing the performance of nomogram in entire TCGA, CGGA, and WCH cohort respectively. LRS, lipid metabolism risk score; ROC, receiver operator characteristic.

The LRS of glioma patients were calculated as follows: LRS= 0.250*GNAI3+0.116*ACACB+0.107*ADCY3+0.084*GLB1+0.038*G6PC3+0.022*PAFAH2-0.001*NEU4-0.010*LCAT-0.019*HADHA-0.051*SGPL1-0.150*ACADSB. Consistently, the protein expression of GNAI3 was higher in the more aggressive high-grade gliomas in the Human Protein Atlas immunohistology study (**Figure S1A**), while that of ACADSB exhibited a reverse trend (**Figure S1B**). Based on the optimal cutoff of LRS, the TCGA-training group was divided into high-risk group and low-risk group (range -2.058-4.738, optimal cutoff 0.947, **Figure S1C**). Otherwise, CGGA (range 2.938-19.828, optimal cutoff 8.352, **Figure S1D**) and WCH (range -0.078-1.244, optimal cutoff 0.553, **Figure S1E**) cohorts were divided into two risk groups based on the same algorithm. In the TCGA cohort, the LRS risk stratification showed profound prognostic value in both the training group and validation group (**Figures 3A, B**). The prognostic capability of LRS was also evaluated in CGGA (**Figure 3C**) and WCH (**Figure 3D**) cohort. Then univariate (**Figure 3E**) and multivariate (**Figure 3F**) Cox regression analysis was conducted to further demonstrated that LRS was an independent prognostic predictor (HR 1.44, 95%CI 1.11-1.88, p=0.025) among other clinical and pathological factors in the TCGA cohort. In TCGA validation group (**Figure 4A**), the AUC of 1-, 2-, 3-year survival ROCs were 0.849, 0.908, 0.901 respectively. The AUCs of 1-, 2-, 3-year survival were 0.764, 0.847, 0.889 in the CGGA cohort (**Figure 4B**), while in the WCH cohort (**Figure 4C**), they were 0.761, 0.708, 0.662, which indicated LRS was a robust and strong prognostic predictor. In TCGA validation group, when compared with other clinicopathological variables, we found 3-year survival ROC curve of LRS possessed maximum AUC (**Figure 4D**), which was consistent in CGGA (**Figure S2A**). In WCH, the AUC of the LRS was 0.688, which was smaller than IDH mutation and 1p19q codeletion (0.747 and 0.709, **Figure S2B**).

### Nomogram construction for predicting prognosis of glioma patients

In the multivariate Cox analysis combining the LRS and clinicopathological factors, LRS, along with WHO grade, IDH mutation status, and radiotherapy were found to be independent prognostic factors. To find out if the LRS and these variables could be integrated to accurately predict the survival risks of glioma patients, a nomogram was established to predict 1-, 2-, and 3- years survival rates in the TCGA, CGGA, and WCH cohort (**Figures 4E**, **S2C, D**). Compared with LRS alone, the multivariate nomogram showed superiority in predicting prognosis (C-index 0.835 vs. 0.852 in TCGA; 0.774 vs. 0.788 in CGGA), but in the WCH cohort, the C-index of the multivariate model was 0.710 and 0.717 for LRS alone. The 1-, 2-, 3-year calibration curves for nomograms demonstrated the accuracy of the multivariate nomograms in predicting the survival time of patients in three cohorts (**Figures 4F–H**).

### Associations between LRS and the clinical, pathological, and molecular characteristics of gliomas

**Figure 5:**
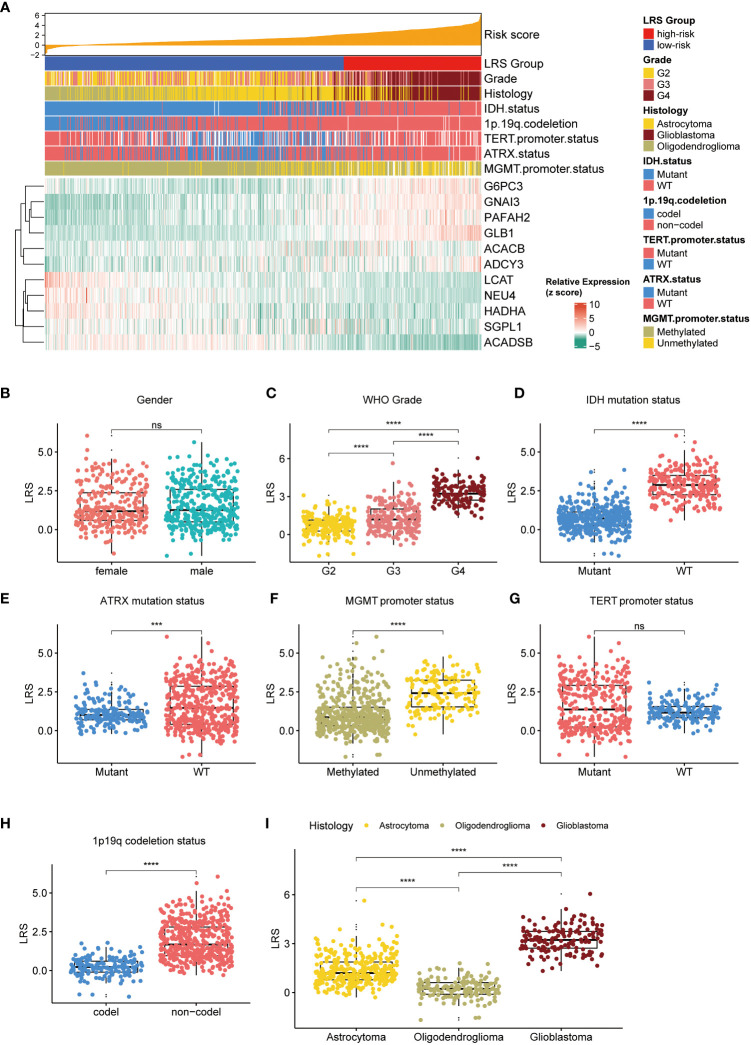
Relationship between LRS and clinicopathological variables. **(A)**, waterfall plot depicted relationship between LRS, 11prgnostic LMRGs and clinicopathological variables; Relationship between LRS and clinicopathological variables including gender **(B)**, WHO grade **(C)**, IDH mutation status **(D)**, ATRX mutation status **(E)**, MGMT promoter status **(F)**, TERT promoter status **(G)**, 1p19q codeletion status **(H)**, and histology **(I)**. LRS, lipid metabolism risk score; LMRG, lipid metabolism-related gene. ns, non-significant. *** p<0.001, **** p<0.0001 in the current study.

To understand if high or low LRS was connected to certain clinical, pathological, and molecular features of the gliomas, we investigated the association between the LRS and available data in the TCGA cohort. First, we analyzed the correlations between LRS and clinicopathological parameters (**Figure 5A**). The results implied that LRS was significantly related to WHO grade, IDH mutation status, ATRX mutation status, MGMT promoter methylated status, TERT promoter mutation status, 1p19q codeletion status, and histology, but not sex (**Figures 5B–I**).

**Figure 6:**
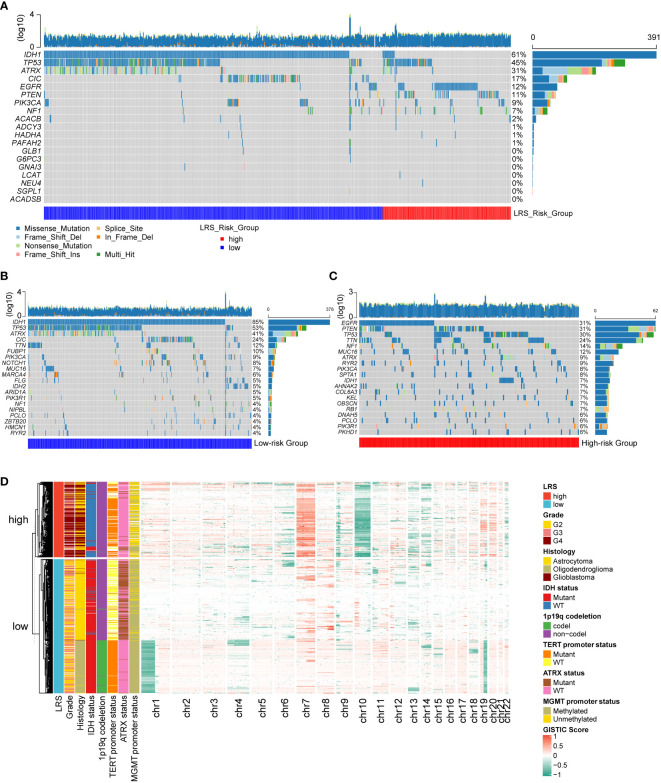
Genetic alterations of prognostic LMRGs in gliomas. **(A)**, somatic mutations of 11 prognostic LMRGs; Top 20 genes with highest degree of mutations in low-risk group **(B)** and high-risk group **(C)**; **(D)** CNV analysis of low- and high-risk group. LMRG, lipid metabolism-related gene; CNV, copy number variation.

Next, we evaluated the relationship between LRS and the genetic aberrations of gliomas. We found that the 11 prognostic LMRGs had low frequency of genetic alterations compared with the common alterations in gliomas (n=644) like IDH-1 mutation, TP53 mutation, and EGFR alterations (**Figure 6A**). By comparing the top 20 genes with highest frequency of somatic mutations in low- (n=441) and high-risk (n=203) group, we found that higher frequency of IDH, ATRX, and CIC mutations in gliomas with low LRS risk (**Figure 6B**), while mutations of EGFR, PTEN, and NF1 were more frequent in the high-risk group (**Figure 6C**). Results of CNV analysis showed amplification of chromosome 7 and loss of chromosome 10 in the high-risk group, while loss of chromosome 1p and 19q were common in the low-risk group (**Figure 6D**).

### Functional enrichment analysis according to risk groups

**Figure 7:**
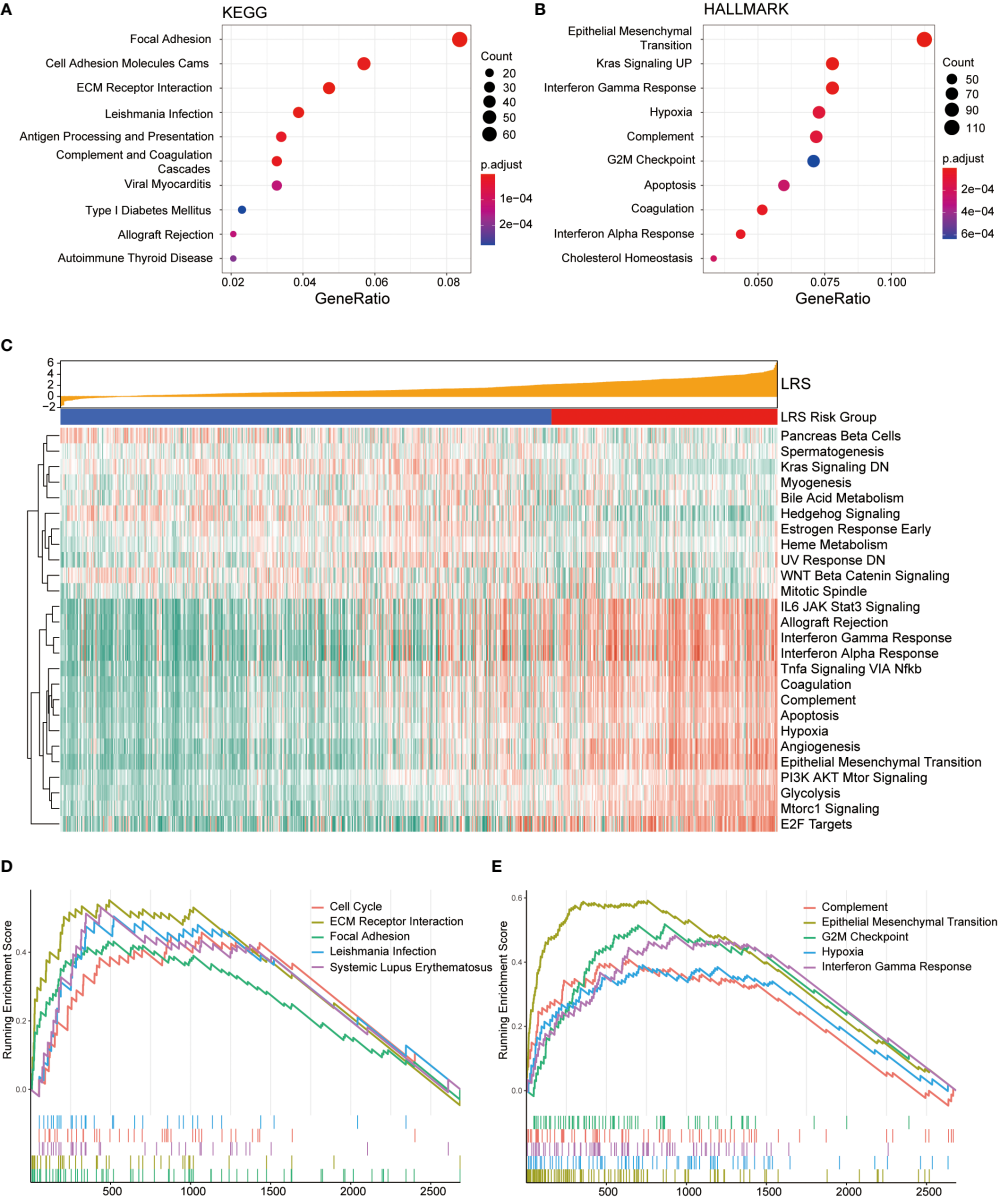
Functional enrichment analysis of DEGs from low- and high-risk groups. Top 10 over-represented KEGG pathways **(A)** and Hallmark pathways **(B)**; **(C)**, Association between LRS and expression of Hallmark gene sets based on GSVA; Top 5 KEGG **(D)** and Hallmark gene sets **(E)** with highest normalized enrichment score based on GSEA.

To further explore the biological differences between LRS high-risk and low-risk groups, we investigated the functional annotation of differentially expressed genes (DEGs). The top 10 KEGG pathways and Hallmark pathways were shown in **Figures 7A, B**. In KEGG pathways, DEGs were significantly associated with focal adhesion, immune-related pathways like antigen processing and presentation, allograft rejection. While in Hallmark gene sets, DEGs were enriched in EMT, KRAS signaling, G2/M checkpoint, Hypoxia, and interferon-related pathways, which were more tightly related to oncogenesis.

GSVA was conducted to present the pathway functional status differences between two groups. In the Hallmark gene sets (**Figure 7C**), the results indicated that relevant pathways with high expression level in the high-risk group were IL6-JAK-STAT3 signaling, allograft rejection, interferon gamma response, interferon alpha response, TNFα signaling, coagulation, complement, apoptosis, hypoxia, angiogenesis, epithelial-mesenchymal transition (EMT), which were mainly involved in immune and inflammatory response and tumor progression. GSVA based on KEGG pathway gene sets suggests activation of anabolic pathways including DNA replication, mismatch repair, and amino/nucleotide sugar metabolism in the high-risk group (**Figure S3A**).

GSEA was also performed in the DEGs between the low- and high-risk groups to explore the cancer-related KEGG pathway and cancer Hallmarks based on LRS. The top 5 KEGG pathways and Hallmark pathways were list in **Figures 7D, E**. Among the KEGG pathway, the DEGs were significantly enriched in cell cycle and focal adhesion in the cancer Hallmark gene sets, they showed remarkable enrichment in the G2/M checkpoint and EMT hallmarks.

In addition, GO enrichment analysis indicated that DEGs enriched in neurogenesis, cell-cell signaling, biological adhesion, neuron differentiation in GO biological pathways (GO-BP, **Figure S3B**), signaling receptor binding, protein containing complex binding, calcium ion binding, passive transmembrane transporter activity in GO molecular function (GO-MF, **Figure S3C**), and synapse, intrinsic component of plasma membrane, neuron projection, plasma membrane region, cell surface in GO cellular compartment (GO-CC, **Figure S3D**).

### Impact of LRS on immune TME landscape

**Figure 8:**
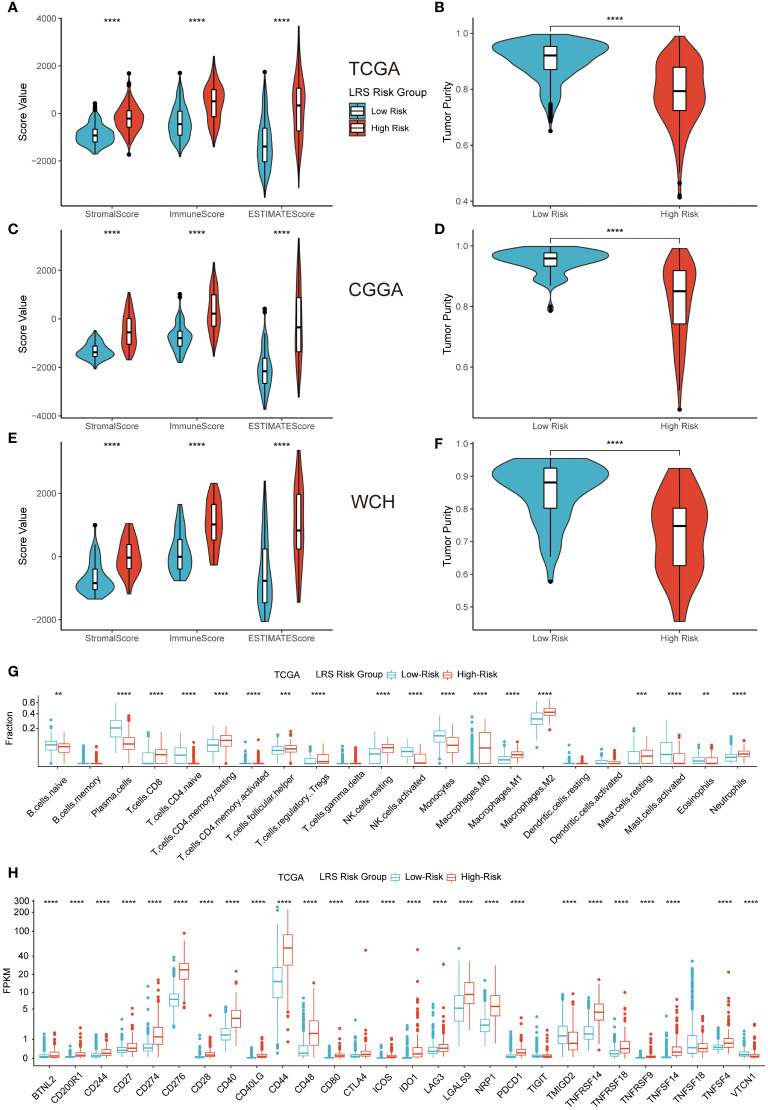
Impact of LRS on TME immune landscape. ESTIMATE **(A–F)** and CIBERSORTx **(G)** algorithm for evaluating TME immune characteristics of glioma; **(H)**, Expression level of ICPs in low- and high-risk groups in TCGA cohort. ** p<0.01, *** p<0.001, **** p<0.0001 in the current study.

To explore the relationship between LRS and TME immune landscape, ESTIMATE and CIBERSORTx algorithm that based on gene expression data were firstly applied. The results of ESTIMATE in three cohorts consistently indicated that LRS values were significantly correlated with stromal score, immune score, and ESTIMATE score, which meant high-risk group had higher stromal score, immune score, and ESTIMATE score (**Figures 8A, C, E**). Meantime, the results revealed that tumor purity of high-risk group was significantly lower than low-risk group (**Figures 8B, D, F**). Together, these results suggest higher infiltration of non-tumor stromal and immune cells in the LRS high-risk gliomas.

Analysis from CIBERSORTx indicated that high-risk group had higher proportions of CD8 T cells, resting memory CD4 T cells, T follicular helper cells, regulatory T cells (Tregs), resting NK cells, all kinds of macrophages (M0, M1, M2), resting mast cells, and neutrophils. While low-risk group had higher proportions of plasma cells, naïve CD4 cells, activated memory CD4 cells, activated NK cells, monocytes, activated mast cells, and eosinophils (**Figure 8G**). Correlation analysis between the predicted immune cells fractions and expression of prognostic LMRGs revealed prominent correlations of M2 and plasma cells with the expression of GNAI3, GLB1, and ACADSB. (**Figure S4A**).

Representative immune checkpoint molecules including PD-1 (CD279), PD-L1 (CD274), CTLA-4 (CD276) were also investigated for association with LRS in entire TCGA cohort. The results of pairwise correlation relationship between the expression of ICPs and LRS were highly correlated (**Figure 8H**). Elevated LRS was significantly related to higher ICP expression levels in the vast majority of ICPs. Similar results were replicated in CGGA and WCH cohort (**Figures S4B, C**).

The above results indicated that compared with low-risk group, high-risk group a more immunosuppressive and complicated TME.

### Therapeutic response prediction based on LMRG risk group

**Figure 9:**
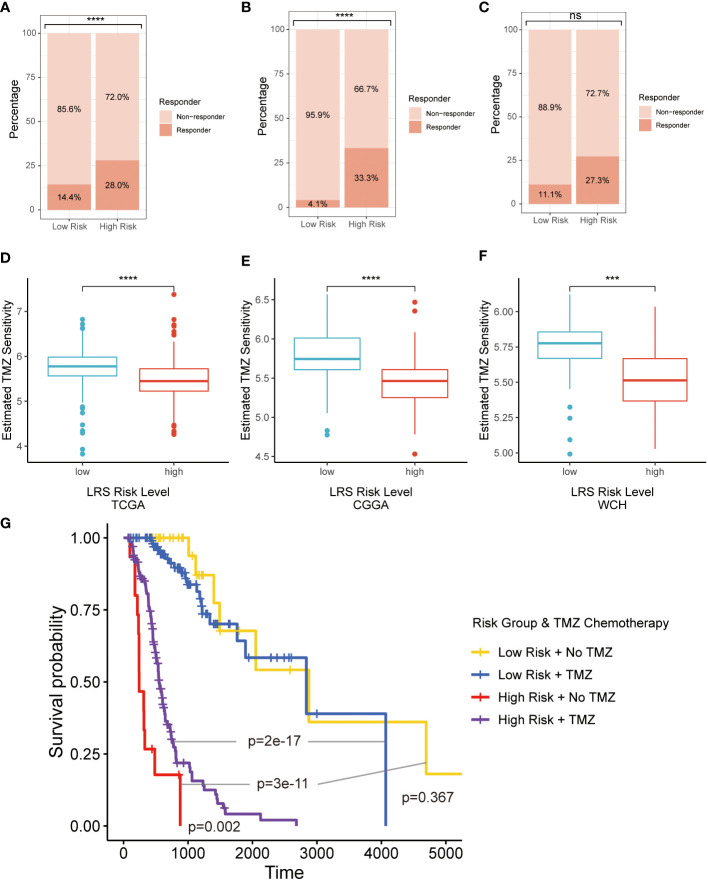
Therapeutic response prediction based on LMRG risk group. **(A–C)**, TIDE algorithm for predicting therapeutic response of ICB for glioma patients in three cohorts; **(D–F)**, pRRophetic for evaluating the sensitivity of TMZ for glioma treatment in three cohorts; **(G)**. K-M curves for evaluating prognosis patients in LRS low- and high-risk groups receiving radiotherapy with or without TMZ. LMRG, lipid metabolism-related gene; TIDE, Tumor Immune Dysfunction and Exclusion; ICB, immune checkpoint blocker; K-M, Kaplan-Mier; TMZ, temozolomide. ns, non-significant. *** p<0.001, **** p<0.0001 in the current study.

Based on the results of the TIDE algorithm, we found that compared with the low-risk group, the high-risk group possessed a larger proportion of glioma patients who were potentially more sensitive to ICBs in TCGA (**Figure 9A**) and CGGA (**Figure 9B**) cohorts. Same results emerged in WCH cohort (**Figure 9C**), but there existed no significant difference between the two risk groups.

The second-generation alkylating agent temozolomide (TMZ) is the first-line chemotherapeutic drug for glioma patients, which has shown good performance in prolonging OS and delaying disease progression (27). pRRophetic was implemented to assess the sensitivity of TMZ for glioma treatment based on different risk groups. The results indicated that glioma patients in low-risk group was more sensitive to the treatment of TMZ in all 3 cohorts (**Figures 9D–F**). In retrospective Kaplan-Meier analysis of patients in the TCGA cohort who have received radiotherapy, the LRS low-risk gliomas had significantly better outcome compared to the LRS high-risk gliomas. However, the survival benefit from TMZ was significant in patients with LRS high-risk gliomas but not LRS low-risk gliomas (**Figure 9G**).

## Discussion

Transcriptome is a useful and practical tool in researching metabolism-related issues in spite of the difficulty in measuring and investigating them in a direct way. In the current study, by utilizing RNA-seq data from clinical specimens, we introduced an innovative lipid metabolism-related gene signatures in gliomas and elucidated their function in prognosis prediction and the association with TME immune characteristics. Taking advantage of a huge amount of bioinformation, we successfully depicted the prognostic and immune landscape in glioma patients based on LMRGs. These reflected that lipid metabolism reprograming in gliomas not only affect disease progression, but also participated in the remolding of TME immune populations.

A large amount of preclinical and clinical evidence over the years indicated that hyperactive lipid metabolism not only feed malignant cancer cells with abundant material and energy supply, but also participate in a crosstalk with oncogenic signaling pathways (28). In gliomas, lipid metabolism reprogramming plays important roles in tumorigenesis, progression, and drug-resistance (29, 30). As shown in the LRS formula, the expression of GNAI3, ACACB, ADCY3, and ACADSB emerged as the most important LMRGs that contribute to the risk signature of gliomas patients. Meanwhile, the LRS risk level were found to be associated with differential epithelial/mesenchymal cellular state in the functional enrichment analysis. These results led us to hypothesize on a model in which reprogrammed lipid metabolism supply a large amount of free fatty acids (FFA), acyl-CoA, acetyl-CoA, and other downstream products to promote malignant behaviors of tumor cells in the high-LRS gliomas (**Figure S5**). Consistent with this hypothesis, Shakya et al. observed elevated level of polyunsaturated fatty acids in the glioma stem cells compared to the non-stem cells, which coincided with reduced lipid droplet accumulation and neutral lipids (31). Additionally, our GSVA and TMZ sensitivity prediction results also suggest that the lipid metabolic shift with increasing LRS may contribute to the progression and drug-resistance of gliomas by promoting synthesis, saturation and plasticity of cell membrane, as well as histone acetylation of the cancer genome (32, 33). Indeed, the Kaplan-Meier analysis here found that under adjuvant radiotherapy plus TMZ, the LRS low-risk gliomas had significantly better outcome than those in the LRS high-risk group. However, when comparing patients receiving adjuvant radiotherapy with or without TMZ, no significant difference was observed in LRS low-risk group. This could be attributed to retrospective nature of the analysis where baseline risk differences may exist between patients receiving adjuvant TMZ or not for less aggressive gliomas, for example, comorbidity status and extent of resection. Therefore, prospective randomized trials are need to elucidate the confusion.

The impact of reprogrammed lipid metabolism is not restricted to cancer cells, but also implies changes in other cell types in TME (34). Specifically, accumulation of FFA in the TME could provide survival advantage to cells that favor fatty acids as energy source (19). Besides, growing evidence has shown that lipid metabolism causes transient generation or accumulation of toxic metabolites which leads to endoplasmic reticulum stress and then regulate the epigenetic modification of immune checkpoints (35). Lipid metabolism can also influence exosome transportation of checkpoints and the degradation of checkpoints (35). In addition, immune cells in TME like B cells (36), macrophages (37), infiltrating T cells (38), and NK cells (39) are all undergo the above-mentioned regulation caused by lipid metabolism, which is beneficial for tumor cells escaping immunological surveillance. Cancer-associated fibroblasts also involve in lipid secretion for cancer cell catabolism and lipid signaling (40). The associations between the tumor immune and lipid metabolism have been extensively studied in many cancers, including lung adenocarcinoma (41), colorectal carcinoma (42), hepatocellular carcinoma (43), etc. However, different to previously identified LMRGs in other cancers, the functional implications of gliomas LMRGs in the FFA metabolism suggest that the FFA enriched TME could be the potential primary culprit for the immunosuppressive TME of gliomas (**Supplementary Figure 5**).

In the current study, we found the representative ICP genes expression PD-1, PD-L1, CTLA-4 were different in specific LMRG risk group and the difference was significantly related to prognosis. For various tumor types, the PD-1/PD-L1 axis is the major speed-limiting step of the anti-cancer immune response (44). In most cases, tumors with high expression of PD-1 or PD-L1 have poor prognosis, but part of them are sensitive to ICI inhibitor, the representative example is triple negative breast cancer (45). Upregulation of ICP gene expression could attenuate the activity of immune cells, allowing cancer cells to escape immunosurveillance and improve their ability in surviving and metastasis (46). The treatments of anti-PD-1/PD-L1 therapy target gliomas have started in recent years, which most focus in GBM (47). Although there are many ongoing clinical trials exploring the efficacy of PD-1/PD-L1 blockades like pembrolizumab and nivolumab in primary or recurrent GBM, breakthroughs have not been achieved in improving prognosis due to the relatively immunosuppressive TME in central nervous system (6). Consequently, screening and targeting specific TME immunological patterns of GBMs or other malignant gliomas that are sensitive to immunotherapies is crucial in future research. Although, we have enough glioma samples and classify them into different subgroups with specific TME immune characteristics based on lipid metabolism-related genes enrichment, most of these patients in TCGA and CGGA have not received immunotherapy and we cannot make further analysis about drug susceptibility of these blockers. However, considering the immunosuppressive features of aberrant lipid metabolism, it might become therapeutic target in various tumors, especially in the organs which have high metabolic rate of lipid. Cutting off lipid supplies and blocking downstream lipid metabolism are the most practical ways. For example, the combined treatment of low toxic AMP-activated protein kinase (AMPK) activator and fatty acid synthase inhibitor synergistically impeded ovarian cancer peritoneal metastases (48).

There are still some limitations in our study. First, part of patients was excluded from the research due to incomplete clinic data, which might lead to selection bias. Second, due to the small number of patients with recurrent glioma from TCGA and CGGA, we only included primary gliomas in the research. This might weak the reliability of current conclusion. Third, because ICIs have not applied on a large scale in glioma patients, there lacks the relevant data about immunotherapy, so we can’t make analysis to accurately predict its therapeutic effects and responses on glioma patients. Fourth, it is not clear whether lipid metabolism or LMRGs are the core driver of remodeling TME, and experimental validation is needed to address this issue based on reasonable design.

## Conclusion

In the current study, we found that the risk model based upon expression program of lipid metabolism-related genes could effectively predict prognosis in patients with glioma. A lipid-metabolism risk score based on the model could divide glioma patients into different groups with distinct clinical, pathological, and TME immune characteristics. Our results indicate that immunotherapy could be beneficial to certain glioma patients with specific lipid metabolism profile.

## Data availability statement

Data from TCGA and CGGA cohorts can be accessed *via* corresponding data portals. The sequencing data generated in this study are available at the Genome Sequence Archive for Humans (https://ngdc.cncb.ac.cn/gsa-human/, accession number: HRA002839, access link: https://ngdc.cncb.ac.cn/gsa-human/s/XRStoK4w).

## Ethics statement

The studies involving human participants were reviewed and approved by Ethical Committee of Sichuan University. Written informed consent to participate in this study was provided by the participants’ legal guardian/next of kin.

## Author contributions

Study design: JL, SZ, YL. Data retrieve: JL, SC, YY, ZW. Statistical Analysis: SZ, MZ. Result interpretation: JL, SZ, TL. Writing-original draft: All authors. Writing-revise: YL, SZ. All authors contributed to the article and approved the submitted version.

## Supplementary material

Supplementary Figure 1
**(A, B)**, Representative immunohistochemical staining for GNAI3 **(A)** and ACADSB **(B)** in high- and low-grade glioma from the Human Protein Atlas (https://www.proteinatlas.org/). **(C–E)**, Distributions and optimal cutoffs of LRS in TCGA-training group **(C)**, CGGA **(D)**, and WCH **(E)** cohorts. TCGA, The cancer Genome Atlas; CGGA, Chinese Glioma Genome Atlas; WCH, West China Hospital; LRS, lipid metabolism risk score.

Supplementary Figure 2
**(A)**, Overall survival prediction based on clinicopathological variables in CGGA cohort; **(B, C)**, Nomogram construction in CGGA and WCH cohorts. CGGA, Chinese Glioma Genome Atlas; WCH, West China Hospital.

Supplementary Figure 3
**(A)**, GSVA in low- and high-risk groups based on KEGG pathway. **(B-D)**, GO enrichment analysis. Abbreviation: GSVA, gene set variation analysis; KEGG, Kyoto Encyclopedia of Genes and Genomes; GO, gene ontology; BP, biological process; MF, molecular function; CC, cellular component.

Supplementary Figure 4
**(A)**, LMRGs affected TME immune cells population; **(B, C)**, Expression level of ICPs in low- and high-risk groups in CGGA and WCH cohorts. LMRG, lipid metabolism-related gene; TME, tumor microenvironment; ICP, immune checkpoint CGGA, Chinese Glioma Genome Atlas; WCH, West China Hospital.

Supplementary Figure 5 Hypothetical mechanism models for the functional implications of LRS. The elevated G-couple protein GNAI3 induces production of cAMP by Adenylate Cyclase 3 (ADCY3), which promotes degradation of triglycerides (TG) and diglycerides (DG) release of free fatty acids (FFA) from lipid droplets (LD). The FFA were converted to acyl-CoA, acetyl-CoA, and other downstream products to facilitate malignant behaviors of high LRS gliomas, or enter the tumor-microenvironment (TME) to convey an immunosuppressive niche.

Supplementary Table 1 Lipid metabolism -related pathway retrieved from MSigDB. MSigDB, Molecular Signature Database.

Supplementary Table 2 Lipid metabolism-related genes and corresponding lipid metabolism pathway.

Supplementary Table 3 DEGs between gliomas and normal brains in TCGA cohort. DEG, different expression gene; TCGA, The cancer Genome Atlas.
