# Supplementary figures and images for: Lipid metabolism-related gene signature predicts prognosis and depicts tumor microenvironment immune landscape in gliomas
Source: Front Immunol. 2023 Feb 13;14:1021678. doi: 10.3389/fimmu.2023.1021678 (PMC9968762; doi:10.3389/fimmu.2023.1021678)

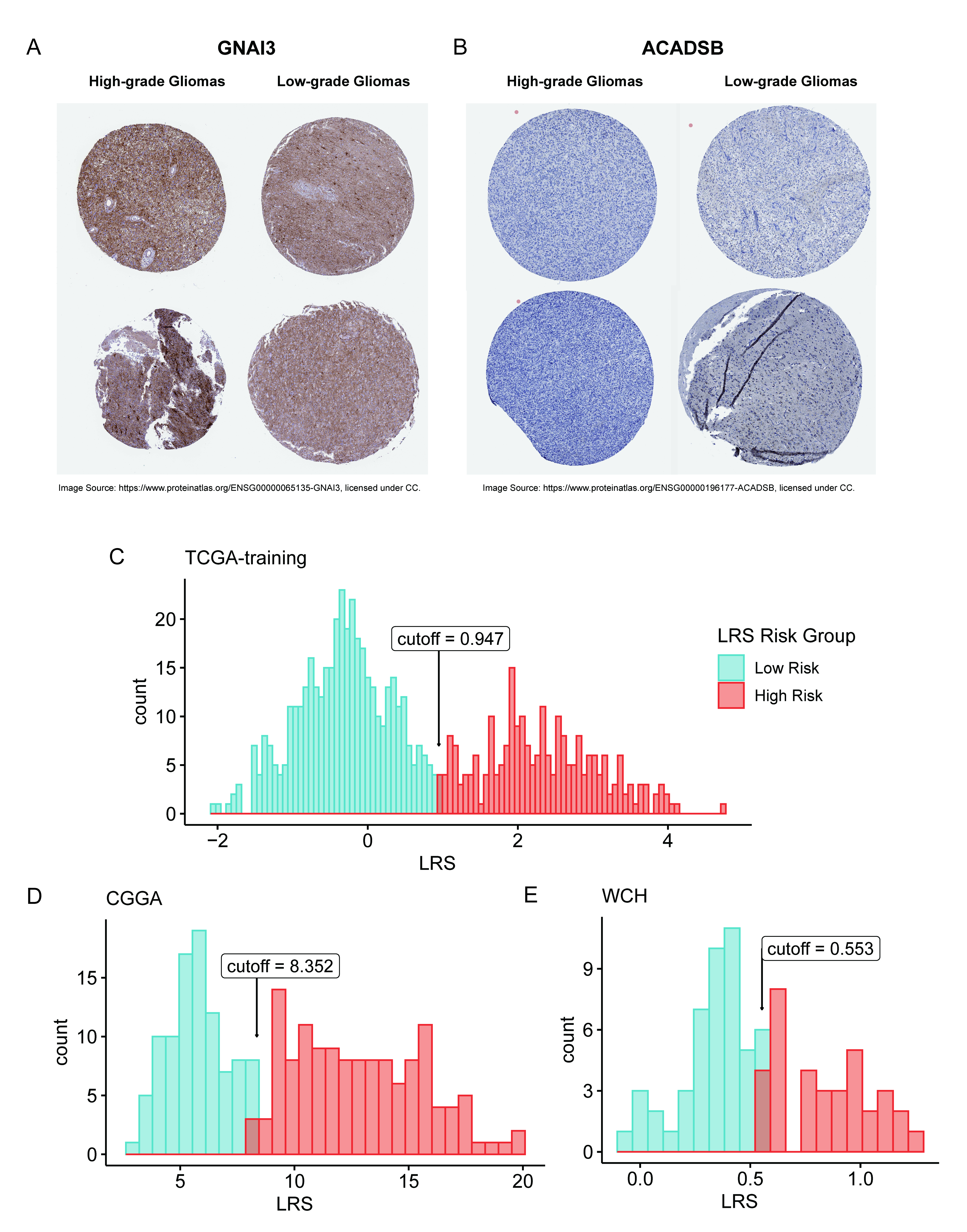

Supplement: Supplementary Figure 1 — (A, B), Representative immunohistochemical staining for GNAI3 (A) and ACADSB (B) in high- and low-grade glioma from the Human Protein Atlas (https://www.proteinatlas.org/). (C–E), Distributions and optimal cutoffs of LRS in TCGA-training group (C), CGGA (D), and WCH (E) cohorts. TCGA, The cancer Genome Atlas; CGGA, Chinese Glioma Genome Atlas; WCH, West China Hospital; LRS, lipid metabolism risk score. [file Image_1.tif]

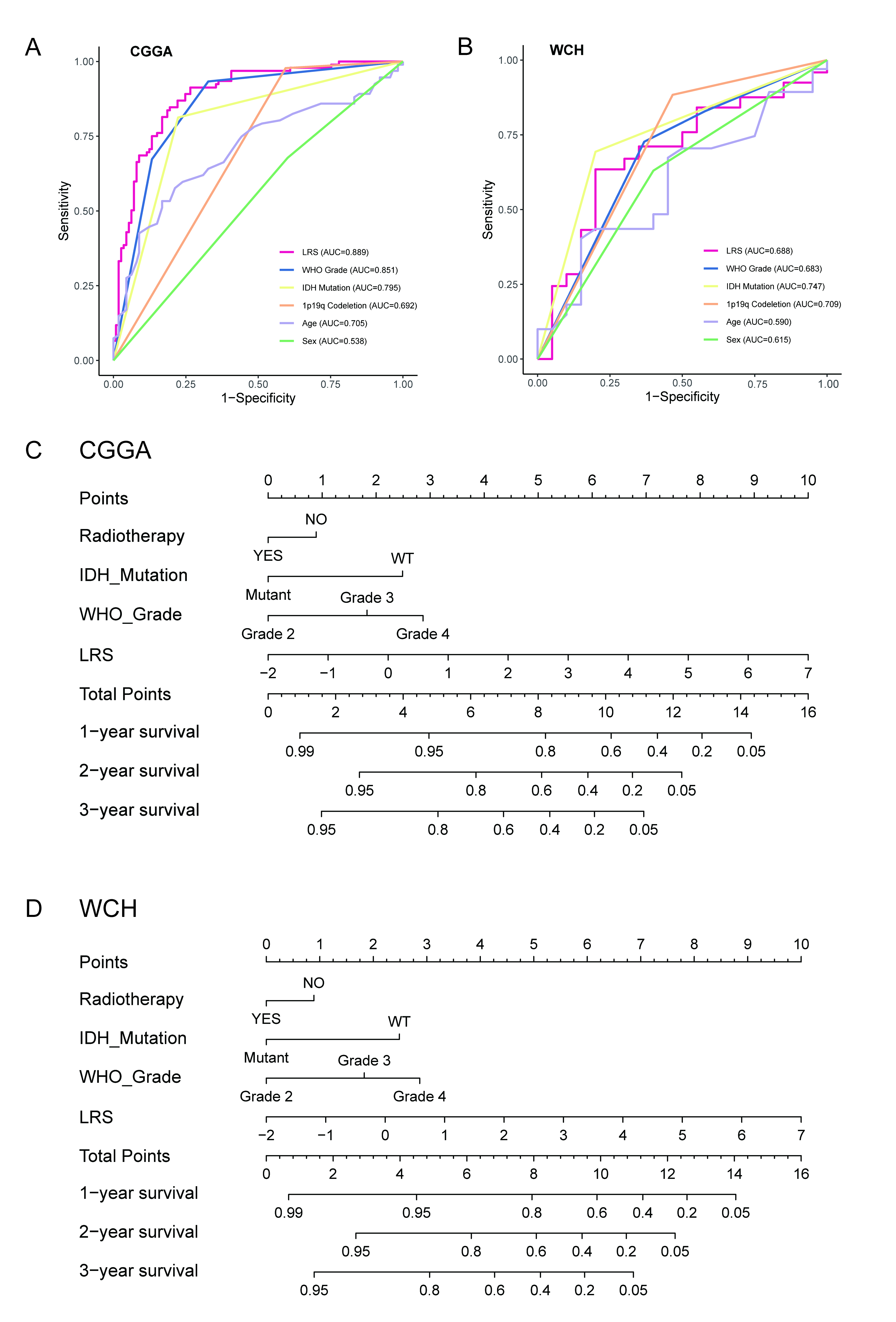

Supplement: Supplementary Figure 2 — (A), Overall survival prediction based on clinicopathological variables in CGGA cohort; (B, C), Nomogram construction in CGGA and WCH cohorts. CGGA, Chinese Glioma Genome Atlas; WCH, West China Hospital. [file Image_2.tif]

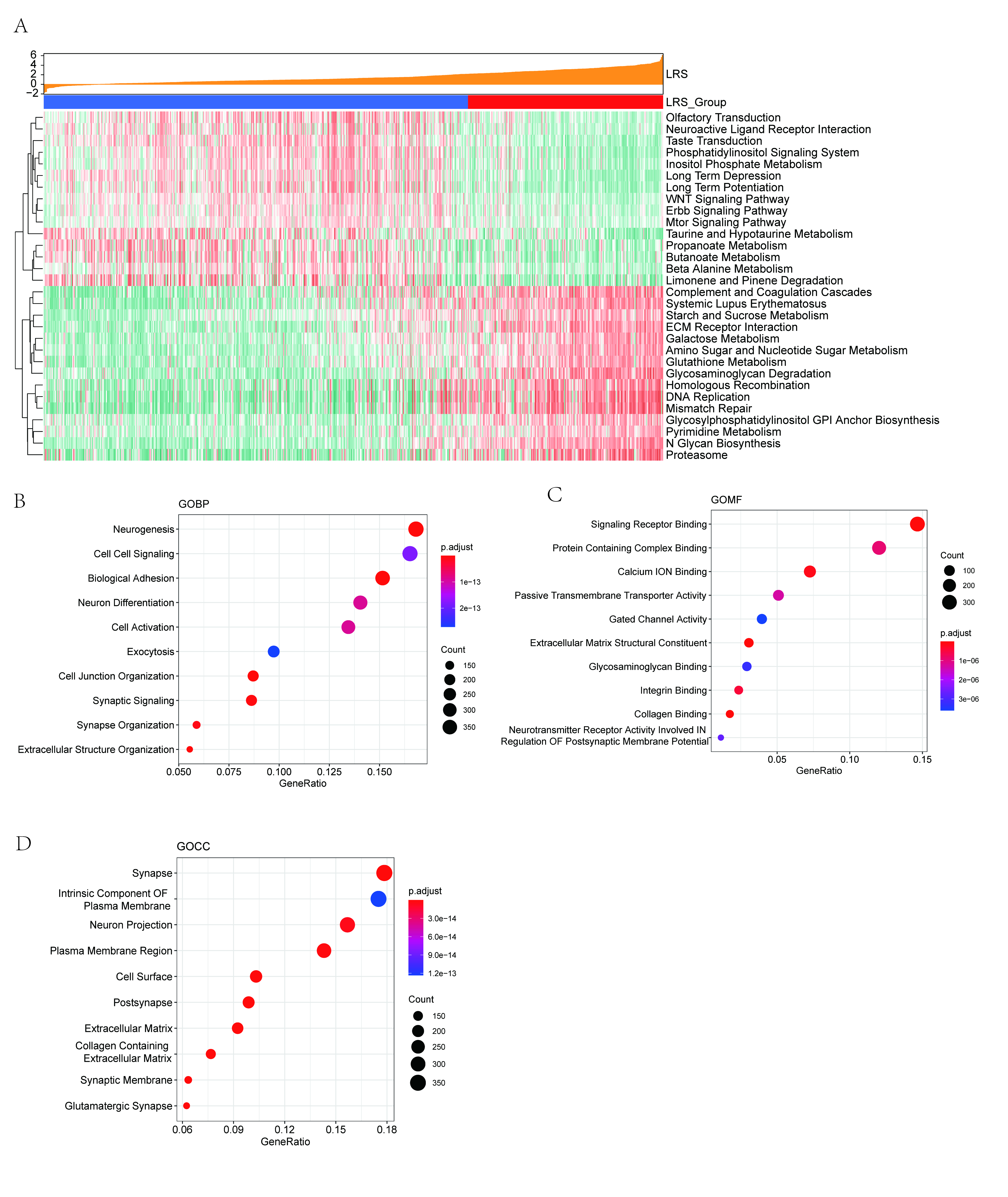

Supplement: Supplementary Figure 3 — (A), GSVA in low- and high-risk groups based on KEGG pathway. (B-D), GO enrichment analysis. Abbreviation: GSVA, gene set variation analysis; KEGG, Kyoto Encyclopedia of Genes and Genomes; GO, gene ontology; BP, biological process; MF, molecular function; CC, cellular component. [file Image_3.tif]

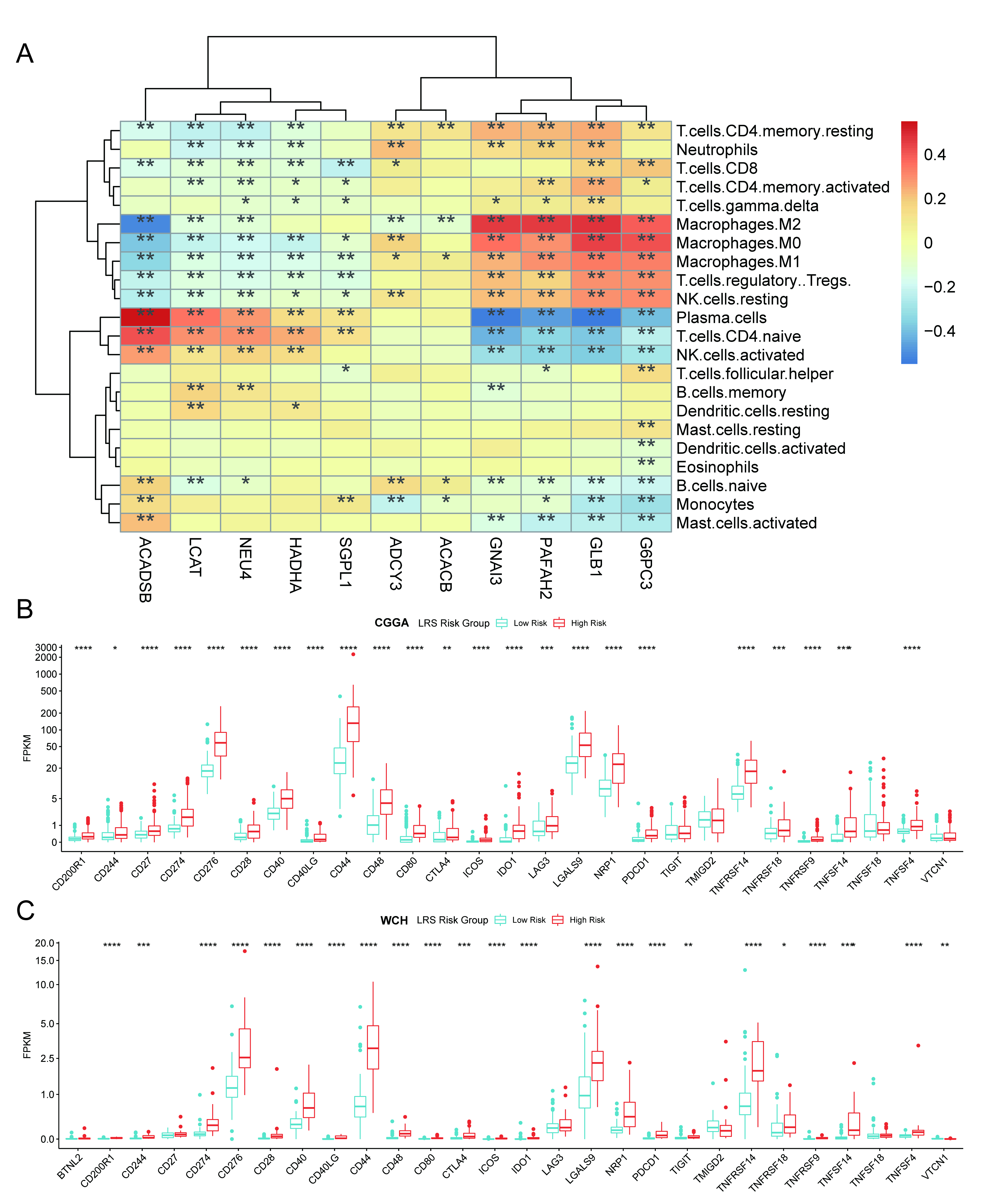

Supplement: Supplementary Figure 4 — (A), LMRGs affected TME immune cells population; (B, C), Expression level of ICPs in low- and high-risk groups in CGGA and WCH cohorts. LMRG, lipid metabolism-related gene; TME, tumor microenvironment; ICP, immune checkpoint CGGA, Chinese Glioma Genome Atlas; WCH, West China Hospital. [file Image_4.tif]

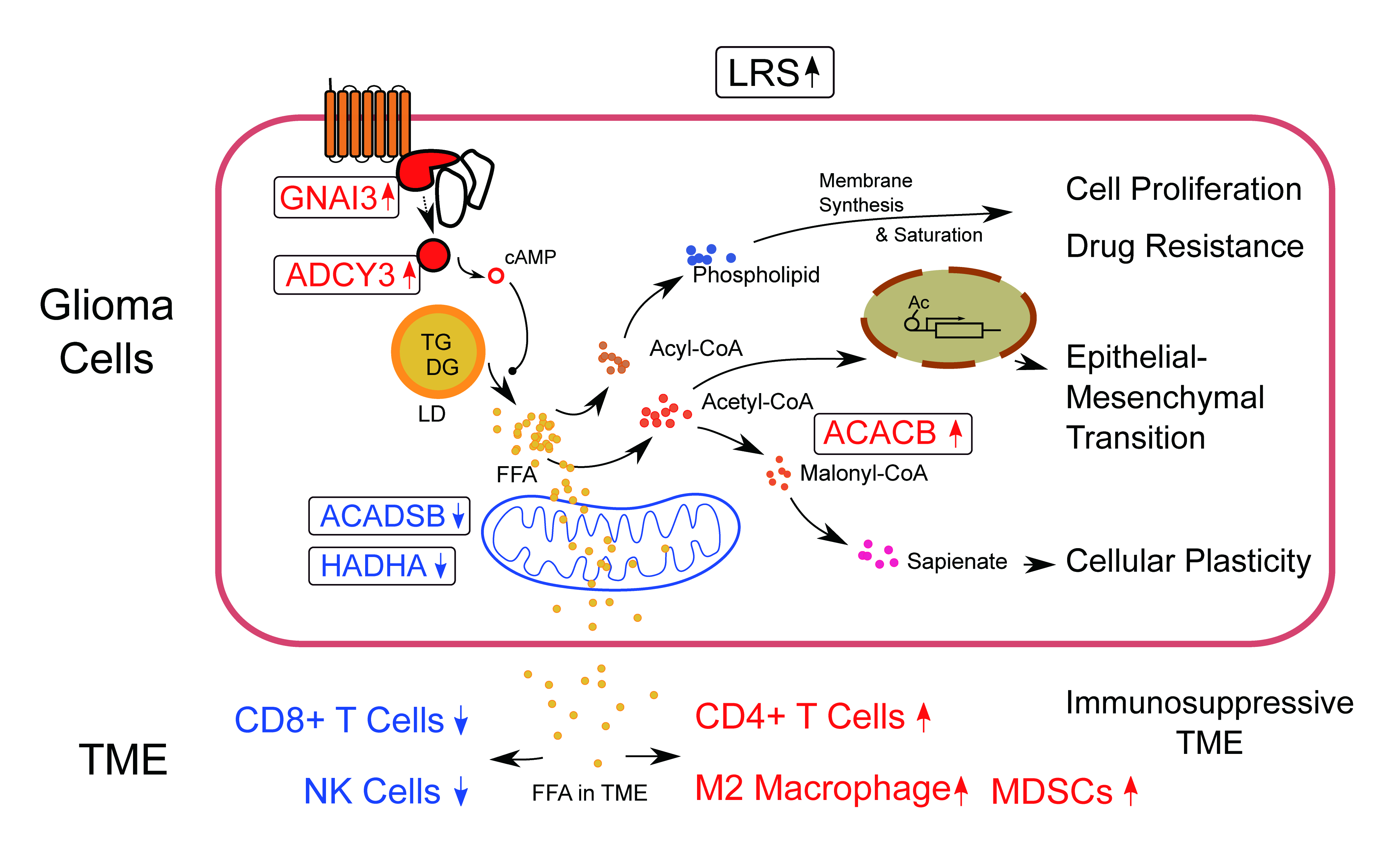

Supplement: Supplementary Figure 5 — Hypothetical mechanism models for the functional implications of LRS. The elevated G-couple protein GNAI3 induces production of cAMP by Adenylate Cyclase 3 (ADCY3), which promotes degradation of triglycerides (TG) and diglycerides (DG) release of free fatty acids (FFA) from lipid droplets (LD). The FFA were converted to acyl-CoA, acetyl-CoA, and other downstream products to facilitate malignant behaviors of high LRS gliomas, or enter the tumor-microenvironment (TME) to convey an immunosuppressive niche. [file Image_5.tif]
